# Primary Retroperitoneal Seminoma—An Uncommon Presentation With Significant Implications

**DOI:** 10.1155/cris/4376859

**Published:** 2024-12-18

**Authors:** S. Sain, S. Tripathi, N. Bakshi, S. A. P. Das, S. Nundy

**Affiliations:** ^1^Institute of Surgical Gastroenterology, GI and HPB Oncosurgery and Liver Transplant, Sir Ganga Ram Hospital, New Delhi, India; ^2^Department of Pathology (Histopathology Division), Sir Ganga Ram Hospital, New Delhi, India

**Keywords:** BEP chemotherapy, burn-out phenomenon, extragonadal germ cell tumor, primary retroperitoneal seminoma

## Abstract

**Background:** Primary retroperitoneal seminoma is an exceedingly rare type of germ cell tumor, accounting for less than 5% of all such tumors. These tumors are typically large at presentation due to their slow growth and the nonspecific nature of symptoms, which often leads to delayed diagnosis.

**Case Presentation:** A 40-year-old male presented with intermittent abdominal pain and a palpable lump in the right paraumbilical region. Ultrasonography revealed a large retroperitoneal mass. Fine needle aspiration cytology confirmed the diagnosis of poorly differentiated malignant tumor, for which he was evaluated with CT-angiogram of the abdomen and FDG PET-CT scans, which showed a large retroperitoneal mass. The patient, then, had a surgical resection of the mass, with postoperative histopathological and immunohistochemical diagnosis of primary retroperitoneal seminoma, and then underwent three cycles of BEP chemotherapy. Scrotal ultrasonography showed no testicular abnormalities, obviating the need for orchiectomy. FDG PET showed a complete response following treatment completion. Postoperative management included routine monitoring of tumor markers and follow-up imaging, which showed a complete response.

**Conclusion:** This case highlights the diagnostic and therapeutic challenges of primary retroperitoneal seminoma. A multidisciplinary approach, including accurate histopathological diagnosis and a combination of chemotherapy and surgery, is essential for optimal management. Early diagnosis and tailored treatment strategies significantly improve patient outcomes.

## 1. Introduction

Primary retroperitoneal seminoma is an extremely rare tumor, comprising about less than 5% of all germ cell cancers. These tumors can occur almost anywhere along the midline, typically presenting as mediastinal, pineal, or retroperitoneal masses. The retroperitoneum accounts for 30%–40% of primary extragonadal seminoma cases, making it the second most common site after the mediastinum, which represents 60%–70% of cases. The designation “primary” indicates that these tumors are associated with morphologically normal testes [1].

The diagnostic evaluation of primary retroperitoneal seminomas is challenging due to their rarity and nonspecific presenting symptoms such as abdominal or back pain, and weight loss. Imaging and histopathological investigations play a crucial role in differentiating these tumors from other potential diagnoses. High-frequency ultrasonography of the testes is essential for distinguishing primary extragonadal tumors from secondary metastases of regressed testicular tumors.

Historically, treatment for primary retroperitoneal seminomas involved surgical excision followed by radiotherapy or chemotherapy, tailored to the tumor's extent and stage. Recent advancements favor a combined therapeutic approach, where initial chemotherapy is followed by surgical resection of the residual tumor mass. This strategy has improved outcomes, yielding higher rates of complete tumor clearance and reduced perioperative complications.

This case report presents a unique instance of primary retroperitoneal seminoma, highlighting diagnostic complexities, therapeutic strategies, and the importance of a multidisciplinary approach in managing this rare entity. By comparing our findings with existing literature, we aim to contribute to the growing body of knowledge and provide insights that may aid in the early diagnosis and effective management of similar cases in the future.

## 2. Case Discussion

### 2.1. Preoperative Course

A 40-year-old male, with no known comorbidities, presented with intermittent, nonradiating right-sided abdominal pain over 4–5 months. He also reported a palpable lump in the same region. Initial ultrasonography of the abdomen revealed a large heterogeneous mass (10.8 × 9.28 × 8.6 cm, 456 cc volume) in the right paraumbilical region. Endoscopic ultrasound (EUS) was performed to characterize the mass and also to get an fine needle aspiration cytology (FNAC) done. The EUS confirmed a large mass (10.2 × 9 cm) adjacent to the mesenteric vessels.

**Figure 1:**
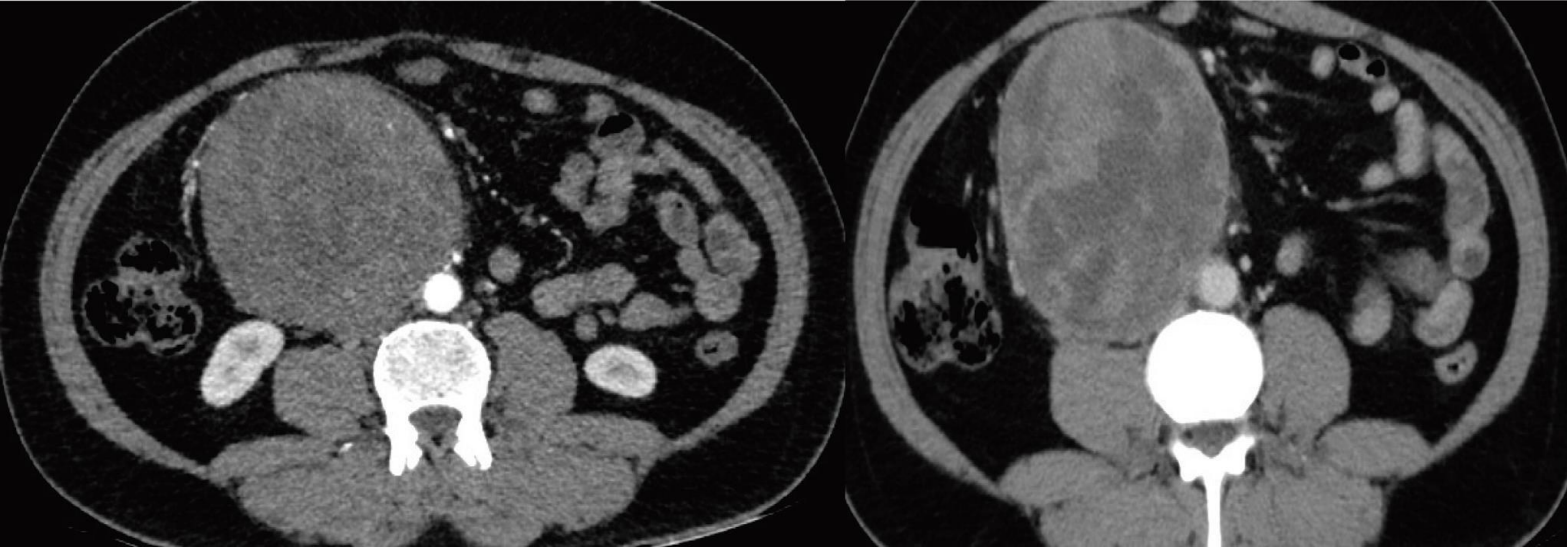
CT-angiogram.

CT angiography of the abdomen revealed a large, well-defined, rounded hypodense mass (10.4 × 10.1 × 12.3 cm) in the right peritoneal cavity (Figure 1). The angiogram also showed a normal caliber abdominal aorta with normal division into the common iliac arteries, and defined ostia of the celiac axis, superior mesenteric artery (SMA), bilateral renal arteries, and inferior mesenteric artery (IMA). The jejunal branches of the SMA were splayed over the mesenteric mass. The superior mesenteric vein united with the retropancreatic splenic vein to form the main portal vein, which had normal intrahepatic branches. The mass had multiple hyperdense areas and specks of calcification, with inhomogeneous peripheral enhancement and central irregular hypodense areas suggesting necrosis. It closely abutted the third part of the duodenum, extended inferiorly to the pelvic inlet, and significantly compressed the inferior vena cava (IVC). The above findings suggested differential diagnoses of mesenteric sarcoma or gastrointestinal stromal tumor. The relationship of the mass to these major vessels and organs was critical in planning the surgical approach.

FNAC of the mass showed abundant necrotic tissue with a few interspersed tumor cells, but no lymph node tissue was identified. Immunohistochemistry (IHC) revealed the tumor cells expressed SALL-4, CD117, Vimentin, PLAP, and OCT3/4, with weak focal Pancytokeratin expression, suggesting a poorly differentiated mesenteric malignant tumor.

**Figure 2:**
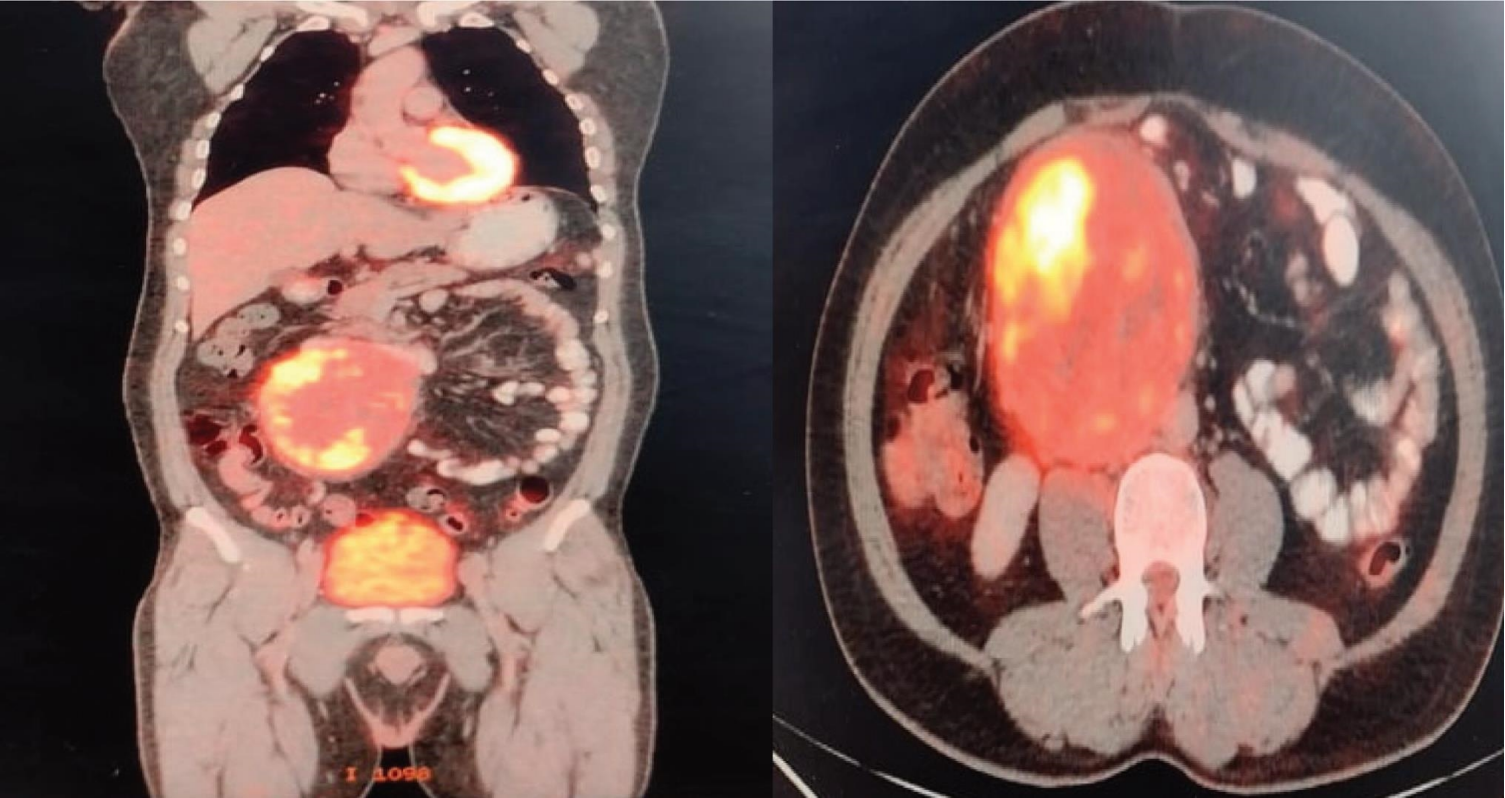
PET-CT.

A whole-body FDG PET-CT scan showed a large, FDG-avid, heterogeneously enhancing mass in the right abdomen, with an SUV value of 13.85 (Figure 2). The scan indicated compression of the IVC and the third part of the duodenum, with the mass closely abutting the abdominal aorta. The FDG avidity, as indicated by the SUV value, suggested high metabolic activity and potential malignancy. Additionally, there were a few FDG-avid enlarged adjacent mesenteric lymph nodes, likely indicating metastasis. No other FDG-avid lesions were seen in the body, providing a focused area for surgical intervention.

Preoperative routine blood tests, including complete blood count (CBC), liver function tests, kidney function tests, and CA 19-9 and CEA, were within normal limits. Based on these findings, a clinical diagnosis of a retroperitoneal mass was made, and radical surgery with curative intent was planned transperitoneally.

### 2.2. Operative Course

**Figure 3:**
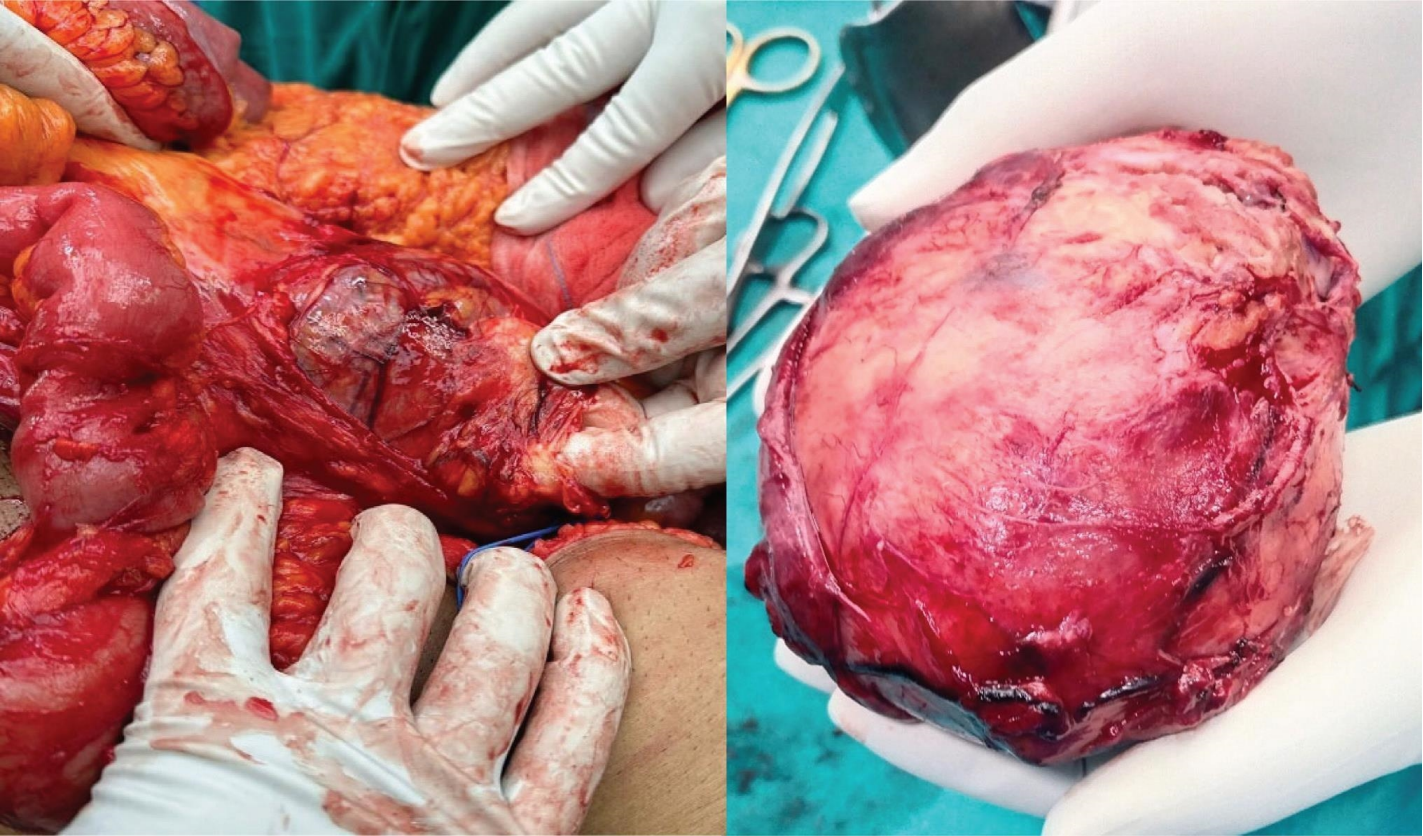
Excised lesion.

An exploratory laparotomy was performed through a midline incision. A large retroperitoneal mass, ~10 × 12 cm, was found abutting the IVC and aorta, displacing the entire small bowel to the left side of the abdomen (Figure 3). The mass was densely adherent to the IVC, aorta, and the third and fourth parts of the duodenum. Mobilization of the right colon was carried out to expose the mass, which was then meticulously separated from all surrounding adhesions.

During adhesiolysis, a perforation in the duodenum was discovered. This necessitated a primary repair of the duodenal perforation, followed by pyloric exclusion and Roux-en-Y gastrojejunostomy with jejunojejunostomy. Additionally, a feeding jejunostomy and cholecystectomy were performed. Throughout the surgery and the immediate postoperative period, the patient remained hemodynamically stable.

On postoperative day 5, an oral Gastrografin follow-through study was conducted, which showed no leakage of contrast. The patient was gradually transitioned to a soft diet and was discharged on postoperative day 10 in a stable condition.

### 2.3. Pathological Examination

**Figure 4:**
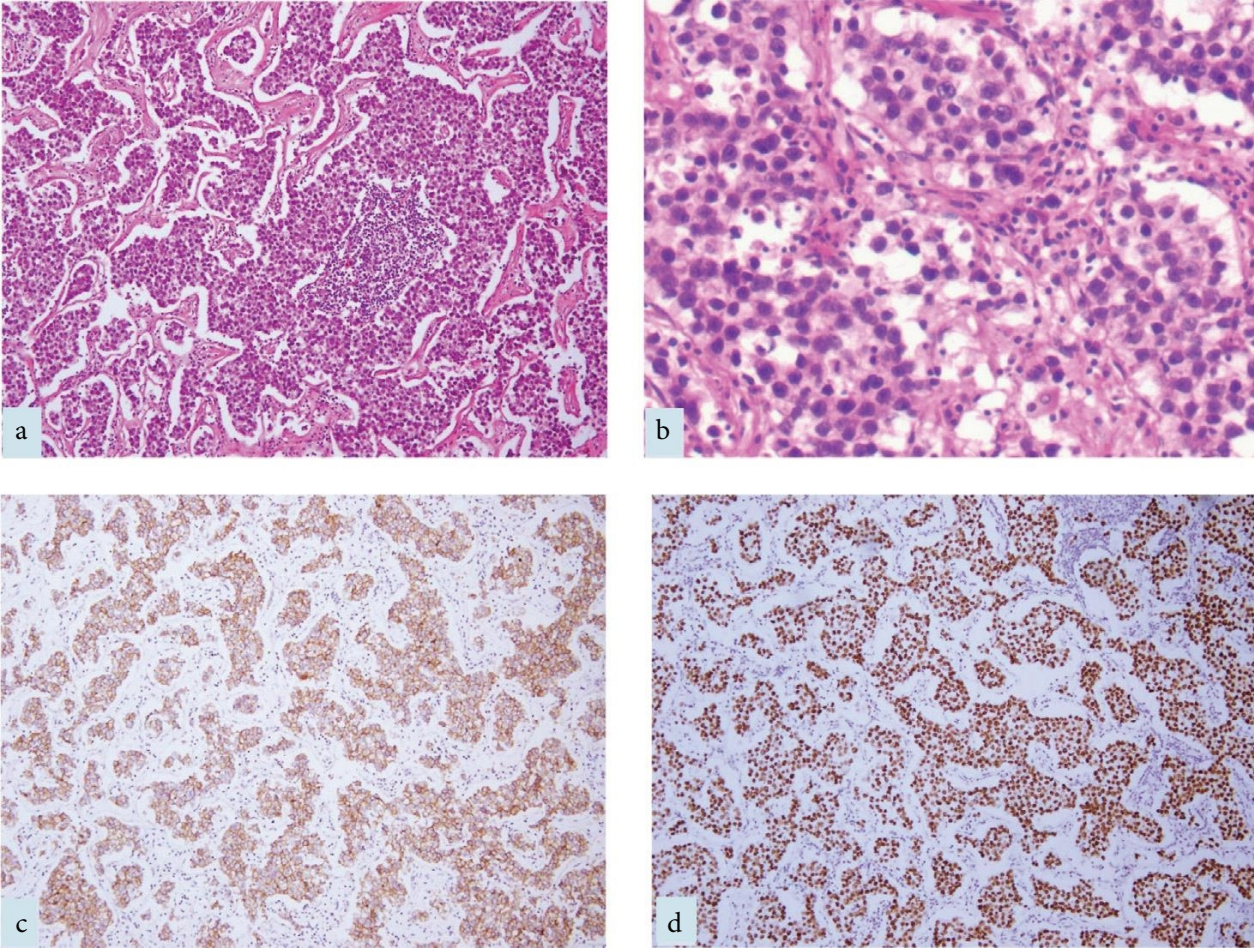
(a) Low power image showing dyscohesive nests and sheets of tumor cells, with dense lymphocytic infiltrate in the intervening fibrovascular stroma (HE, 4x). (b) Tumor cells are round to polygonal and exhibit moderate nuclear pleomorphism, vesicular chromatin with prominent nucleoli and pale eosinophilic cytoplasm (HE, 20x). (c) Tumor cells are diffusely positive for CD117 (IHC, 10x). (d) Diffuse OCT3/4 expression (IHC, 10x). IHC, immunohistochemistry.

Figure 4 describes the histopathological imaging for the patient. Gross examination of the resected specimen revealed a lobulated, encapsulated mass measuring 12 × 10 × 9 cm, with areas of necrosis and congestion, and a focal breach in the capsule. Microscopic examination showed extensive necrotic areas with intervening viable tumor tissue arranged in a lobular pattern. The tumor cells were organized in nests and solid sheets, displaying moderately pleomorphic, ovoid to polygonal nuclei with vesicular chromatin, prominent nucleoli, and pale eosinophilic to clear cytoplasm. The stroma was densely infiltrated by lymphocytes, with focal tiny lymphoid aggregates.

IHC showed diffuse expression of SALL4, CD117, OCT3/4, and PLAP in the tumor cells, with focal, weak cytokeratin (CK) staining. CD30, Glypican 3, LCA, and synaptophysin were negative, confirming the absence of other germ cell components. The final pathology diagnosis was extragonadal germ cell tumor (EGCT)—seminoma. The pathologist recommended a thorough examination of the bilateral testes to rule out a primary gonadal origin.

### 2.4. Postoperative Course

Postoperative assessment included evaluating the patient's tumor markers: *β*HCG (beta-human chorionic gonadotropin), LDH (lactate dehydrogenase), and *α*FP (alpha-fetoprotein), in the light of the histopathological diagnosis. The LDH level was elevated at 483 IU/L, while *β*HCG and *α*FP levels were within normal ranges. A thorough clinical re-examination of both testes was conducted, and ultrasonography of the scrotum revealed that the bilateral testes were normal in size and position, with no scars or focal lesions. The right testis measured 3.6 × 2.7 × 1.8 cm (volume 9.6 mL), and the left testis measured 3.1 × 1.9 × 1.9 cm (volume 9 mL).

Following these assessments, the patient was referred to a medical oncologist, who recommended three cycles of chemotherapy with BEP (bleomycin, etoposide, and cisplatin), which was started after POD-21, as per the standard protocol followed at our center. The patient completed the full course of chemotherapy. Subsequent follow-up, 3-month postoperation, included a repeat FDG PET scan, which showed a complete response to the treatment. The patient continues to be monitored according to standard follow-up protocols, which include clinical follow-up every 3 months in OPD and abdominal and pelvic CT scan every 6 months, for the first 3 years, postoperation.

## 3. Discussion

Primary retroperitoneal seminoma is an exceedingly rare malignancy, accounting for about 4.4% of all retroperitoneal primary malignancies. These tumors are often large and palpable at presentation due to their slow growth in a spacious area. The nonspecific nature of their symptoms, such as abdominal pain and the presence of a lump, often leads to delayed diagnosis. In our case, a 40-year-old male presented with intermittent abdominal pain and a palpable lump, findings consistent with other reports where patients typically exhibit symptoms like abdominal mass, back pain, and weight loss.

During the fifth week of embryonic development, germ cells migrate from the yolk sac along with epithelial celomic cells and cells from the mesonephros to the dorsal embryonic aspect of the genital ridges [1]. These cells contribute to the formation of primitive sex cords. Through the action of the SRY gene (sex-determining Region Y), the medial aspect of the genital ridges differentiates into Sertoli cells, which remain closely associated with primordial germ cells, eventually forming the testes.

There is ongoing debate regarding the origin of primary EGCTs. One theory suggests that germ cells become trapped in various tissues during their migration from the yolk sac. Another theory proposes a reverse migration of already-formed germ cells to ectopic locations. A contrasting view posits that these retroperitoneal seminomas are actually metastatic deposits from a regressed or “burned out” primary testicular tumor [2].

Our case highlighted several challenges in the preoperative diagnosis and treatment of primary retroperitoneal seminoma. Preoperative differentiation from other retroperitoneal tumors is difficult due to nonspecific clinical, laboratory, and imaging features. FNAC and IHC are crucial in establishing a diagnosis.

Retroperitoneal involvement in seminomas is usually the result of metastasis from a primary testicular tumor via lymphatic channels, and this possibility must be excluded before diagnosing a primary retroperitoneal seminoma. In the absence of a discernible testicular mass, the “burned out” phenomenon should be considered. This phenomenon is defined as the presence of an EGCT without a gross tumor in the testes, but with histological evidence of a previously regressed primary germ cell tumor. High-frequency ultrasonography is essential to detect small testicular lesions that can guide biopsy sites. In our case, thorough examination of the testes with ultrasound showed no abnormalities, ruling out a “burned out” phenomenon. Various histological features like hyalinosis, hemosiderin deposition, and the presence of fibrosis are indicative of an earlier presence of a germ cell tumor of the testis that has completely regressed.

The primary extragonadal origin can be presumed based on specific features: the presence of gonadal tissue in the tumor capsule, encapsulation of the tumor without surrounding lymph node involvement, and a high retroperitoneal lesion with adjacent lymph nodes but not involving lower aortic, iliac, or pelvic lymph nodes [3]. The morphology of primary retroperitoneal seminomas mirrors that of testicular seminomas, but histological diagnosis in the retroperitoneal location requires careful differentiation from other retroperitoneal tumors. Immunohistochemical markers such as PLAP, SALL4, OCT3/4, and CD117 are crucial in this differentiation. PLAP, typically produced by placental syncytiotrophoblasts, is a marker of germ cell neoplasms. OCT3/4 and SALL4 are stem cell transcriptional regulators that maintain pluripotency in embryonic stem cells and germ cells, making them highly specific markers for seminoma.

The management of retroperitoneal seminomas is complicated by their anatomical involvement. In our case, the tumor was densely adherent to the IVC, aorta, and duodenum, complicating surgical excision. Duodenal involvement is particularly challenging and has been associated with significant morbidity and mortality in other cases [4]. In our patient, duodenal perforation postadhesiolysis required complex surgical management, including pyloric exclusion and Roux-en-Y gastrojejunostomy.

Historically, primary surgical treatment for retroperitoneal EGCTs had unsatisfactory outcomes, with high operative mortality rates [5]. However, a combination of initial chemotherapy and subsequent surgical resection has significantly improved disease-free survival rates. In our case, the patient underwent surgical resection followed by three cycles of chemotherapy (BEP regimen), resulting in a complete response on follow-up FDG PET scans.

Given the potential for a “burned out” phenomenon, where primary testicular tumors regress and leave metastases in the retroperitoneum, it is crucial to thoroughly examine the testes and utilize high-frequency ultrasonography in all patients presenting with retroperitoneal masses. In our patient, clinical examination and ultrasonography of the testes showed no abnormalities, consistent with cases of primary retroperitoneal seminomas that present without detectable testicular involvement.

Our case underscores the importance of considering primary retroperitoneal seminoma in the differential diagnosis of retroperitoneal masses, conducting thorough evaluations of the testes, and employing FNAC and IHC for accurate diagnosis. Early histopathological diagnosis is essential to guide the treatment plan, whether it involves neoadjuvant chemotherapy or upfront surgery. Routine monitoring of tumor markers and prompt biopsy in cases of increased markers are recommended to facilitate early diagnosis and improve outcomes.

The treatment of EGCTs remains controversial due to the complexity and variability of these tumors. While some experts advocate for primary surgical resection, others emphasize the importance of neoadjuvant chemotherapy followed by surgery. Historically, primary surgical treatment for retroperitoneal EGCTs has yielded unsatisfactory results, with high operative mortality rates and poor outcomes. However, advancements in chemotherapy have significantly improved disease-free survival rates, making a combination of chemotherapy and surgery the preferred approach in many cases [6]. Initial chemotherapy, typically with regimens such as BEP (bleomycin, etoposide, and cisplatin), helps to shrink the tumor, making surgical resection more manageable and improving overall outcomes.

The decision to perform an orchiectomy in cases of suspected primary retroperitoneal seminoma is also debated. Scholz et al. [7] conducted a histological examination involving ipsilateral/bilateral orchiectomy or testicular biopsy in a series of 26 patients. They found that only seven patients had biopsy-confirmed testicular tumors or intratubular neoplasia. However, in patients with fibrosis and scarred tissue, indicative of a “burned out phenomenon” from a previous testicular primary, routine orchiectomy was recommended by this group [7]. Conversely, Anglade, Chang, and Siroky [8] reported an abnormal testis on ultrasonography in a case of primary retroperitoneal seminoma but found no evidence of a tumor on the contralateral testicle biopsy or ipsilateral orchiectomy specimen.

In a study by Buskirk et al. [9], 12 cases of primary retroperitoneal seminoma treated at the Mayo Clinic were reviewed. Ipsilateral orchiectomy was performed in two patients without any clinical suspicion of testicular tumors. Microscopic examination revealed no testicular tumor or its modifications, and the procedure had no effect on the treatment or progression of the primary retroperitoneal tumor [9]. Based on these findings, our opinion aligns with the view that orchiectomy is not required if the testes are found normal in clinical and radiological evaluations. Preoperative clinical and ultrasonographic evaluations of the testes should be conducted in all cases of retroperitoneal tumors to rule out any testicular involvement.

In our case, scrotal ultrasonography and FDG PET-CT scans showed normal findings, with no evidence of testicular abnormalities. Therefore, we did not perform an orchidectomy. This decision was supported by the absence of any hyperechoic lesions or suspicious findings on imaging. However, it is crucial to note that if a hyperechoic lesion is detected during ultrasonography, an orchidectomy should be considered to exclude the possibility of a “burned out” testicular tumor, which can guide further treatment decisions.

The management of primary retroperitoneal seminoma requires a multidisciplinary approach, involving careful diagnostic evaluation, appropriate use of chemotherapy, and meticulous surgical planning. Routine monitoring of tumor markers and the use of advanced imaging techniques are essential in guiding treatment and improving outcomes. In summary, the combination of neoadjuvant chemotherapy and surgical resection remains the cornerstone of treatment, with orchiectomy reserved for cases with clear indications of testicular involvement.

Considering the age of our patient (41 years old), which falls within the common presentation range for seminoma (35–45 years), appropriate adjuvant therapy was critical. According to the International Germ Cell Cancer Collaborative Group (IGCCCG) prognostic-based staging, our patient was classified in the good prognosis group. This classification was based on the absence of nonpulmonary visceral metastasis, the seminomatous origin of the tumor, and stable tumor markers.

Given these factors, the recommended treatment regimen for patients in this category includes three cycles of bleomycin, etoposide, and platinum (BEP). This regimen aims to reduce tumor burden and improve survival rates effectively. Our patient received the standard three cycles of BEP chemotherapy, in the postoperative period, which do not align with the IGCCCG recommendations for individuals with a good prognosis profile; however, the patient showed complete response, which highlights the importance of an individualized approach should be the standard of care. This approach has been shown to enhance disease-free survival and overall outcomes in patients with similar clinical characteristics.

## 4. Conclusion

Primary retroperitoneal seminoma is a rare and challenging malignancy. This case highlights the importance of thorough diagnostic evaluation, including FNAC and IHC, for accurate diagnosis. The combined approach of chemotherapy followed by surgical resection proved effective, with the patient achieving a complete response. The decision against orchiectomy was based on normal clinical and radiological evaluations of the testes. This case underscores the necessity of a multidisciplinary approach, routine monitoring of tumor markers, and careful assessment of the testes in managing retroperitoneal masses. Early diagnosis and tailored treatment strategies are crucial for improving patient outcomes in primary retroperitoneal seminoma.

## Data Availability

The data that support the findings of this study are available upon request from the corresponding author. The data are not publicly available due to privacy or ethical restrictions.
